# Determination of Flavanones in Orange Juices Obtained from Different Sources by HPLC/DAD

**DOI:** 10.1155/2014/296838

**Published:** 2014-08-07

**Authors:** Lidércia C. R. Cerqueira e Silva, Jorge M. David, Rafael dos S. Q. Borges, Sérgio L. C. Ferreira, Juceni P. David, Pedro S. dos Reis, Roy E. Bruns

**Affiliations:** ^1^Universidade Federal da Bahia, Instituto de Química, 40170-290 Salvador, BA, Brazil; ^2^Universidade Federal da Bahia, Faculdade de Farmácia, 40170-290 Salvador, BA, Brazil; ^3^Universidade Federal do Piauí, Núcleo de Pesquisa em Biodiversidade e Biotecnologia-Biotec, 64202-020 Parnaíba, PI, Brazil; ^4^Universidade Estadual de Campinas, Instituto de Química, 13084-971 Campinas, SP, Brazil

## Abstract

Flavanones (hesperidin, naringenin, naringin, and poncirin) in industrial, hand-squeezed orange juices and from fresh-in-squeeze machines orange juices were determined by HPLC/DAD analysis using a previously described liquid-liquid extraction method. Method validation including the accuracy was performed by using recovery tests. Samples (36) collected from different Brazilian locations and brands were analyzed. Concentrations were determined using an external standard curve. The limits of detection (LOD) and the limits of quantification (LOQ) calculated were 0.0037, 1.87, 0.0147, and 0.0066 mg 100 g^−1^ and 0.0089, 7.84, 0.0302, and 0.0200 mg 100 g^−1^ for naringin, hesperidin, poncirin, and naringenin, respectively. The results demonstrated that hesperidin was present at the highest concentration levels, especially in the industrial orange juices. Its average content and concentration range were 69.85 and 18.80–139.00 mg 100 g^−1^. The other flavanones showed the lowest concentration levels. The average contents and concentration ranges found were 0.019, 0.01–0.30, and 0.12 and 0.1–0.17, 0.13, and 0.01–0.36 mg 100 g^−1^, respectively. The results were also evaluated using the principal component analysis (PCA) multivariate analysis technique which showed that poncirin, naringenin, and naringin were the principal elements that contributed to the variability in the sample concentrations.

## 1. Introduction 

An increase in the consumption of fruits and vegetables is associated with a decrease in the incidence of cardiovascular disease and reduced risks of certain cancers. Citrus fruits and derived products have a beneficial effect on humans [1, 2]. Oranges are a rich source of flavonoids that are bioactive and may protect against age-related diseases. The absorption of orange flavanones may be affected by factors such as processing, plant species, and geographic sources. Additionally, the bioactivity of the absorbed phytochemicals depends on how they are metabolized during absorption [3].

Several analytical methods have been applied to qualitative and quantitative flavonoid determination, especially HPLC in conjunction with diode array detection and mass spectrometry, as in honey [4],* Citrus grandis *[5], grapes and teas [6],* Scutellaria baclensis *[7],* Glycyrrhiza uralensis, G. glabra *and* G. inata *[8], blueberries [9], cultivars of clementine mandarin, satsuma mandarin, hybrid mandarin, navel orange, common orange, and pigmented orange [1],* Citrus* species [10], grape cultivars [11], and orange and grapefruit juices [12].

In the present paper, the flavanone contents (hesperidin, naringin, naringenin, and poncirin) of commercial and homemade orange juice originating from different places in Brazil were evaluated. The data were processed using principal component analysis (PCA) to test homogeneity of the analytical results as well as identify outliers that should be removed from the data set to obtain representative values for flavonoids in Brazilian orange juice. In recent years, this technique has been used for the evaluation and characterization of different kinds of food [13, 14].

## 2. Materials and Methods

### 2.1. Reagents

Standards for HESP (hesperitin-7-O-rutinoside or hesperidin), NARG (naringenin), NAR (naringenin-7-*O*-neohesperidoside or naringin), and PON (isosakuranetin-7-*O*-neohesperidoside or poncirin) were obtained from Sigma (St. Louis). Methanol and acetic acid reagents were obtained from J. T. Baker (Phillipsburg, NJ). All solvents employed were HPLC-grade and ultrapure (Milli-Q) water was used.

### 2.2. Samples of Orange Juice

Six samples (1–6) of orange juice were hand-squeezed (domestic processing), six samples (31–36) were obtained from a fresh-in-squeeze machine, and 24 industrial samples of orange juice were purchased from markets in the Bahia and São Paulo states (Brazil). These 36 samples of orange juice were obtained during 2008-2009 and all belonged to the* Sinensis* variety, a common orange juice source in Brazil.

### 2.3. Preparation of Samples: Extraction of Flavanones

**Figure 1:**
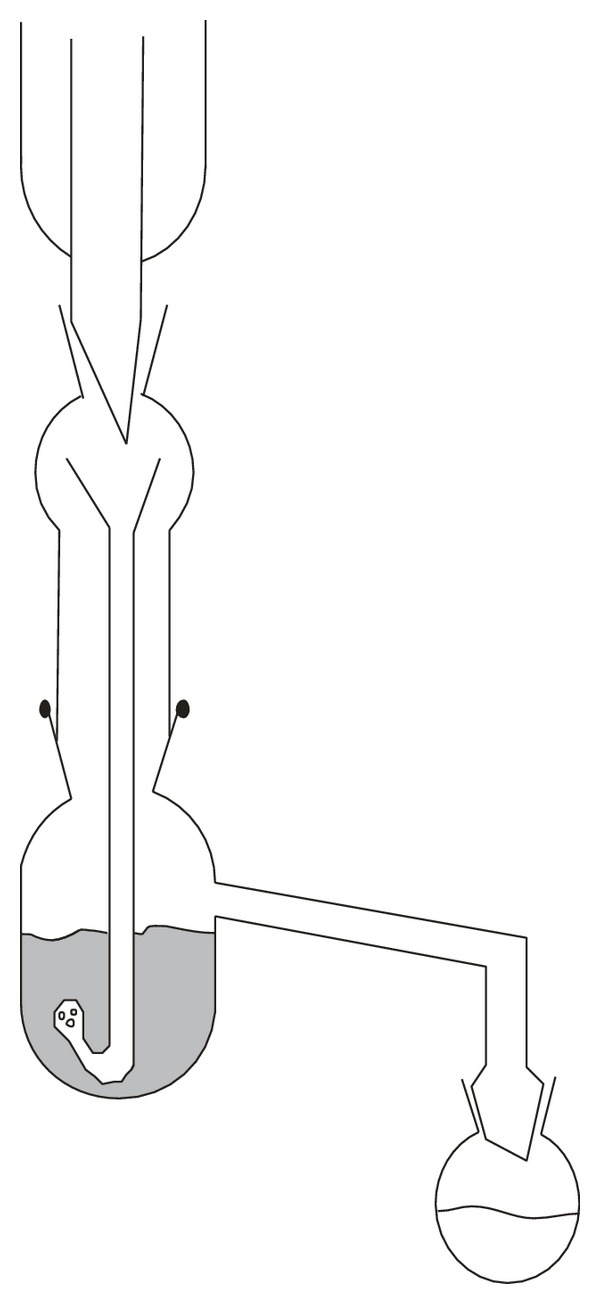
Scheme of liquid-liquid continuous extraction.

The juice samples were prepared using the adapted liquid-liquid continuous extraction shown in Figure 1. Aliquots of 400 g of orange juice were mixed with 300 mL of ethyl acetate (EtOAc). The time of this procedure was 40 min. After extraction, the analyte samples were obtained by evaporation to dryness at 40°C under vacuum. After the residues were filtered through 0.22 *μ*m filters, they were dissolved in 10 mL of methanol before HPLC detection.

### 2.4. HPLC-DAD Analysis

HPLC-DAD analysis was performed on a Shimadzu liquid chromatographic system (Kyoto, Japan) equipped with a Rheodyne injection valve with a 1 *μ*L fixed loop, an SCL-10 AVP pump, and an autosampler. The flow rate was 0.3 mL/min, and a thermostated column oven was used with a reverse-phase C_18_ Purospher STAR tracer 3 *μ*m (55 × 4 mm) (Merck, Germany) column with sample and column temperatures of 5 and 25°C, respectively. Compounds were monitored at 240 and 280 nm using a UV-Vis photodiode array detector (Shimadzu model SPD-MD), and UV spectra were recorded from 200 to 400 nm and were controlled by a GPC/LC solution workstation (version 1.21 SP1).

A gradient mobile phase consisting of methanol (solvent A) and acid H_2_O (1.0% acetic acid) (solvent B) was used for HPLC analysis. The conditions were as follows: initial conditions of 20% A for 5 min, followed by an increase to 60% A for 10 min, 80% A for 20 min, and 60% A in the following 40 min, and then back to the initial conditions for 10 min. The total run time was 50 min at 0.3 mL/min. Compounds were identified by comparing their retention times (Rt NAR = 9.0 min; Rt HESP = 15.0 min; Rt PON = 17.0 min; Rt NARG = 22.0) and their UV-vis spectra with corresponding standards. Concentrations were determined using an external standard curve.

### 2.5. Data Analysis

The statistical analysis program Statistic 6.0 (Statistic Inc., USA) was used to treat the data for basic statistical parameters and principal component analysis (PCA). PCA was applied to data that had been previously autoscaled. Each principal component is an eigenvector of the correlation matrix of the standardized original data and is a linear combination of the original variables. One-way ANOVA was used to estimate significant differences for the calculated data means.

## 3. Results and Discussion 

### 3.1. Validation Studies

The analytical curves were constructed using standard solutions in the 60–300, 0.01–0.09, 0.05–0.25, and 0.05–0.30 mg 100 g^−1^ ranges for hesperidin, naringin, naringenin, and poncirin, respectively. The analytical curves exhibited excellent linear behavior over the concentration range.

The limits of detection (LOD), calculated as the minimal concentration corresponding to 3.3 × (SD/S), with SD being the standard deviation of the blank and S the slope of the standard curve, were 0.0037, 1.87, 0.0147, and 0.0066 mg 100 g^−1^ for naringin, hesperidin, poncirin, and naringenin, respectively. The limits of quantification (LOQ), calculated as the minimal concentration corresponding to 10 × (SD/S), with SD being the standard deviation of the blank and S the slope of the standard curve, were 0.0089, 7.84, 0.0302, and 0.0200 mg 100 g^−1^, respectively, for naringin, hesperidin, poncirin, and naringenin, respectively.

The calculated values were confirmed with actual analytical values under the established chromatographic conditions. The accuracy of the method was verified in recovery tests using flavanone samples spiked with 60 mg 100 g^−1^ of hesperidin and 0.30 mg 100 g^−1^ of flavanone standards (naringin, naringenin, and poncirin). The test was performed three times for each flavonoid. The mean recoveries were 101, 107, 103, and 98%, for naringin, hesperidin, poncirin, and naringenin, respectively. The coefficients of variation showed good repeatability.

The linearity of the HPLC method was checked in the 60–300 mg 100 g^−1^ range for hesperidin and the 0.01–0.09, 0.05–0.25, and 0.05–0.30 mg 100 g^−1^ ranges for naringin, naringenin, and poncirin, respectively. Calibrations were performed by injection of the standard working solution in triplicate at five different concentrations for each flavanone based on the expected flavanone content ranges in the samples. All standard curves passed through the origin were linear in the concentration ranges expected for the samples and had coefficients of determination ranging from 0.9986 (for naringin) to 0.9999 (for naringenin).

### 3.2. Flavanone Levels in Orange Juice

**Figure 2:**
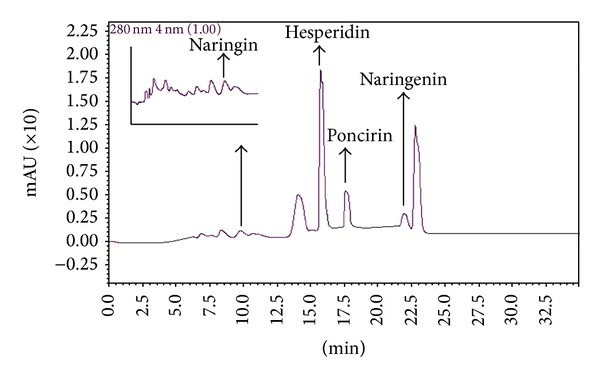
Characteristic HPLC-DAD chromatogram of samples of Brazilian orange juice.

The flavonoids present in the Brazilian orange juices were separated by HPLC and the UV spectra of the different peaks were recorded. The chromatographic conditions allowed for good separation of most of the peaks in the chromatogram. A characteristic HPLC-DAD chromatogram of the EtOAc extract of orange juices recorded at 240 nm is presented in Figure 2. Diode array detection (UV spectra recorded from 200 to 400 nm) allowed characterization of the phenolics, which were mainly flavonoids. Definitive identification of some of the flavanones was performed by spiking experiments with authentic compounds.

The results of the determinations of hesperidin, poncirin, naringin, and naringenin in the 36 orange juice samples are summarized in Table 1. Among these four analytes, hesperidin showed the highest concentration levels. These data are described as 95% confidence intervals. The average content was 69.85 mg 100 g^−1^, and the concentration range found was from 18.80 to 139.00 mg 100 g^−1^. It is worth noting that the industrial orange juices showed high contents of hesperidin (samples 7 to 31). The presence of this compound is important for juice quality, as hesperidin is known to have antioxidant, anti-inflammatory, antiallergic, and immunomodulatory activities, while naringenin is involved in some pharmaceutical metabolism (e.g., dihydropyridines) and decreases the half-life of medicines that are dependent on cytochrome P450 [15–17] and lower blood lipid and cholesterol [18–20].

Naringin, naringenin, and poncirin showed lower concentration levels in the orange juices; the average contents and concentration ranges found were 0.019 and 0.01–0.30, 0.12 and 0.1–0.17, and 0.13 and 0.01–0.36 mg 100 g^−1^, respectively.

The results of the flavanone determinations for the 36 juice samples were analyzed using the multivariate principal component analysis (PCA) technique. This statistical tool was employed to evaluate the extraction tendencies and the quantification of the flavonoids.

**Figure 3:**
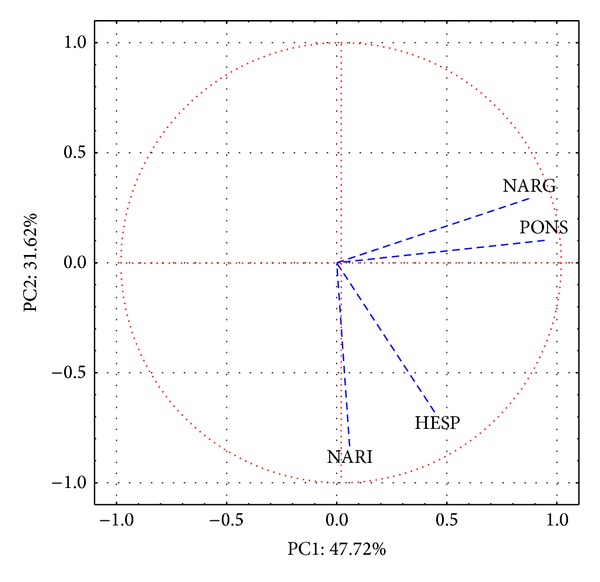
Score plot with PC1 × PC2.

**Figure 4:**
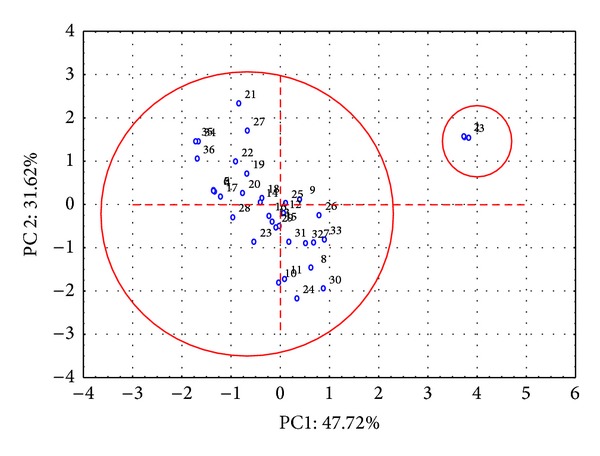
Samples with scores on PC1.

A data matrix was constructed using the flavanone concentrations as columns and the orange juice samples as rows. PCA was performed on autoscaled data.  Figure 3 shows the loadings of the first two principal components. The results of the PCA revealed that the first two principal components explained 79.34% of the total variance. PC1 accounted for 47.72% of the variability in the data, mainly related to the poncirin, naringenin, and naringin concentrations. The score plot (Figure 3) did not show well-defined data clustering of the samples with scores on PC1 (1, 2, and 3, all circled in Figure 4). These samples were hand-squeezed orange juice samples (domestic processing).

Finally, the second principal component (PC2, describing 31.62% of the total variance) was primarily associated with variations in the hesperidin and naringin concentrations. These variables were associated with positive scores on PC2. Thus, the samples corresponding to points located at the top of the score graph in Figure 3 had high contents of these flavanones. According to Gattuso et al. [21], hesperidin is the major flavonoid component of sweet oranges and related species. The distribution of the samples throughout PC2 was relatively uniform, showing that the concentrations of theses flavanones have continuous distributions throughout the juice samples.

## 4. Conclusions

This paper reports determination of the flavanone composition (hesperidin, naringin, naringenin, and poncirin) in orange juice. The samples analyzed indicated that orange juice contains high levels of hesperidin. The PCA technique showed that poncirin, naringenin, and naringin were the principal elements that contributed to the variability in the samples. These results suggest that flavonoid contents are more expressive in industrial samples maybe due to the mechanical process of obtaining the juices.

## Figures and Tables

**Table 1:** Flavanone concentrations in orange juices samples (mg 100 g^−1^)^a^.

Samples	HESP	NARG	NARI	PONS
1	70 ± 1	0.170 ± 0.006	0.016 ± 0.001	0.35 ± 0.02
2	69 ± 1	0.170 ± 0.001	0.016 ± 0.003	0.35 ± 0.01
3	71 ± 2	0.170 ± 0.001	0.016 ± 0.004	0.36 ± 0.01
4	47.5 ± 1	0.12 ± 0.01	0.020 ± 0.001	0.03 ± 0.01
5	46.1 ± 1	0.12 ± 0.01	0.020 ± 0.001	0.03 ± 0.01
6	46.3 ± 0.3	0.12 ± 0.01	0.020 ± 0.001	0.03 ± 0.01
7	109.5 ± 0.2	0.12 ± 0.01	0.020 ± 0.001	0.17 ± 0.01
8	78 ± 3	0.12 ± 0.01	0.030 ± 0.001	0.20 ± 0.01
9	65 ± 1	0.12 ± 0.01	0.020 ± 0.001	0.20 ± 0.01
10	89 ± 1	0.12 ± 0.01	0.030 ± 0.001	0.10 ± 0.01
11	86 ± 2	0.12 ± 0.01	0.030 ± 0.001	0.12 ± 0.01
12	77 ± 1	0.12 ± 0.01	0.020 ± 0.001	0.14 ± 0.01
13	84 ± 1	0.12 ± 0.01	0.020 ± 0.001	0.10 ± 0.01
14	63 ± 1	0.12 ± 0.01	0.020 ± 0.001	0.10 ± 0.01
15	89 ± 1	0.12 ± 0.01	0.020 ± 0.001	0.11 ± 0.01
16	78 ± 1	0.12 ± 0.01	0.020 ± 0.001	0.10 ± 0.01
17	53.07 ± 0.04	0.12 ± 0.01	0.020 ± 0.002	0.010 ± 0.001
18	59 ± 3	0.12 ± 0.01	0.020 ± 0.002	0.11 ± 0.01
19	33 ± 1	0.12 ± 0.01	0.020 ± 0.001	0.11 ± 0.01
20	52 ± 1	0.12 ± 0.01	0.020 ± 0.001	0.07 ± 0.01
21	18.8 ± 0.2	0.12 ± 0.01	0.010 ± 0.001	0.12 ± 0.01
22	20 ± 1	0.12 ± 0.01	0.020 ± 0.001	0.10 ± 0.01
23	45.8 ± 0.4	0.12 ± 0.01	0.030 ± 0.001	0.10 ± 0.01
24	107.1 ± 0.4	0.12 ± 0.01	0.030 ± 0.001	0.12 ± 0.01
25	67.2 ± 2.0	0.12 ± 0.01	0.020 ± 0.001	0.16 ± 0.01
26	139 ± 1	0.12 ± 0.01	0.010 ± 0.001	0.15 ± 0.01
27	47 ± 1	0.12 ± 0.01	0.010 ± 0.001	0.10 ± 0.01
28	75.0 ± 0.1	0.12 ± 0.01	0.020 ± 0.001	0.010 ± 0.001
29	90 ± 1	0.12 ± 0.01	0.020 ± 0.001	0.10 ± 0.01
30	100.2 ± 0.3	0.12 ± 0.01	0.030 ± 0.001	0.20 ± 0.01
31	106 ± 2	0.12 ± 0.01	0.020 ± 0.001	0.11 ± 0.01
32	109 ± 1	0.12 ± 0.01	0.020 ± 0.001	0.15 ± 0.01
33	108 ± 1	0.12 ± 0.01	0.020 ± 0.001	0.20 ± 0.01
34	39 ± 2	0.106 ± 0.002	0.011 ± 0.001	0.06 ± 0.02
35	38 ± 2	0.105 ± 0.002	0.011 ± 0.001	0.06 ± 0.02
36	39 ± 2	0.105 ± 0.003	0.014 ± 0.002	0.06 ± 0.01

^a^Average of three determinations ± interval confidence at 95%.
